# Effects of the “AI-TA” Mobile App With Intelligent Design on Psychological and Related Symptoms of Young Survivors of Breast Cancer: Randomized Controlled Trial

**DOI:** 10.2196/50783

**Published:** 2024-06-04

**Authors:** Lulu Jiang, Jiehui Xu, Yanwei Wu, Yanyan Liu, Xiyi Wang, Yun Hu

**Affiliations:** 1 School of Nursing, Shanghai Jiao Tong University Shanghai China; 2 Renji Hospital Affiliated to Shanghai Jiao Tong University School of Medicine Shanghai China

**Keywords:** mobile app, artificial intelligence, interactivity, breast cancer, psychological symptoms, self-efficacy, social support, quality of life

## Abstract

**Background:**

Young women often face substantial psychological challenges in the initial years following cancer diagnosis, leading to a comparatively lower quality of life than older survivors. While mobile apps have emerged as potential interventions, their effectiveness remains inconclusive due to the diversity in intervention types and variation in follow-up periods. Furthermore, there is a particular dearth of evidence regarding the efficacy of these apps’ intelligent features in addressing psychological distress with these apps.

**Objective:**

This study aims to evaluate the effectiveness of a mobile app with intelligent design called “*AI-TA*” on cancer-related psychological health and ongoing symptoms with a randomized controlled design.

**Methods:**

Women aged 18 to 45 years diagnosed with breast cancer were randomly assigned to the intervention or control group. The intervention was *AI-TA*, which included 2-way web-based follow-up every 2 weeks. Both intention-to-treat (ITT) and per-protocol (PP) analyses employed repeated measurement analysis of variance. The participants’ background features, primary outcomes (psychological distress and frequency, self-efficacy, and social support), and secondary outcomes (quality of life) were measured using multiple instruments at 3 time points (baseline, 1-month intervention, and 3-month intervention).

**Results:**

A total of 124 participants were randomly allocated to the control group (n=62, 50%) or intervention group (n=62, 50%). In total, 92.7% (115/124) of the participants completed the intervention. Significant improvements in psychological symptoms (Memorial Symptom Assessment Scale-Short Form) were observed in the ITT group from baseline to 1-month intervention relative to the control group (ITT vs control: 1.17 vs 1.23; *P*<.001), which persisted at 3-month follow-up (ITT vs control: 0.68 vs 0.91; *P*<.001). Both the ITT and PP groups exhibited greater improvements in self-efficacy (Cancer Behavior Inventory-Brief Version) than the control group at 1-month (ITT vs PP vs control: 82.83 vs 77.12 vs 65.35; *P*<.001) and 3-month intervention (ITT vs PP vs control: 92.83 vs 89.30 vs 85.65; *P*<.001). However, the change in social support (Social Support Rating Scale) did not increase significantly until 3-month intervention (ITT vs control: 50.09 vs 45.10; *P*=.002) (PP vs control: 49.78 vs 45.10; *P*<.001). All groups also experienced beneficial effects on quality of life (Functional Assessment of Cancer Therapy-Breast), which persisted at 3-month follow-up (*P*<.001).

**Conclusions:**

The intelligent mobile app *AI-TA* incorporating intelligent design shows promise for reducing psychological and cancer-related symptoms among young survivors of breast cancer.

**Trial Registration:**

Chinese Clinical Trial Registry ChiCTR2200058823; https://www.chictr.org.cn/showproj.html?proj=151195

## Introduction

### Background

Breast cancer is a significant health concern for women globally, particularly in China, where the incidence and mortality rates have been steadily rising, accounting for 12.2% and 9.6%, respectively, of the total cases in the world [1,2]. In 2020, alone, approximately 416,371 women were newly diagnosed with breast cancer [3]. Moreover, the peak prevalence of breast cancer among Chinese women occurring between 45 and 55 years, which is younger than that of their Western counterparts [2-4].

Life after breast cancer, especially for younger survivors, often entails adverse psychological consequences [5]. Young survivors of breast cancer have greater psychologic morbidity than older women and age-matched women with no cancer history; this includes elevated levels of psychological distress and frequency of persistent disease for at least 2 years after diagnosis [6]. A substantial proportion of younger women experience long-term iatrogenic effects, including fatigue, persistent pain, lymphedema, and infertility, all of which may negatively affect psychological health [7,8]. Other cancer-related symptoms, such as psychosocial maladjustment, have also been reported during both cancer treatment and rehabilitation [9]. A lack of confidence and preparedness to cope with cancer can intensify survivors’ distress, hinder their reintegration into society, reduce their self-efficacy, and cause significant impairment in quality of life [10,11]. Understanding the dynamic demands of young survivors of breast cancer is crucial for providing targeted and culturally sensitive support. Our previous research on young survivors of breast cancer indicated that psychological support is desired early in diagnosis, and there is more focus on information provided during treatment [12]. Thus, recognizing the unique characteristics of young survivors of breast cancer is vital for delivering tailored and comprehensive psychosocial care.

Research indicates that young survivors of breast cancer have more complex and dynamic needs and face challenges related to cultural norms, psychological disturbances, and a decreased quality of survivorship [13]. Web-based programs that leverage the accessibility, availability, and cost-effectiveness of the internet have been widely used in breast cancer interventions [14,15]. However, most programs aimed at improving well-being in survivors of cancer are not tailored to the specific functions, components, or characteristics of the target population. Information is often generalized and looped and does not accurately align resources with individual needs, rendering them ineffective for many patients. This means that there is no coordinated, personalized, or supportive care; rather, there is only a 1-way relationship between programs and patients.

Incorporating artificial intelligence (AI) into interventions offers a promising avenue to address these challenges. AI, as a major component of the internet, can enhance technical interventions and interactions through the use of a sophisticated blend of human-computer and human-human techniques [16]. Specifically, AI algorithms can analyze user input and provide tailored advice or support based on the user’s history and preferences. This personalized interaction increases user engagement and satisfaction, effectively bridging psychological gaps and facilitating a deeper understanding of user needs. At the humanistic level, AI can significantly enhance communication and collaboration. For example, AI-driven platforms can facilitate social support networks or groups, connecting individuals with similar interests or experiences. This approach is particularly useful in therapeutic contexts or web intervention. In health care, AI can analyze patient data in real time to provide up-to-date, personalized health recommendations or alerts, empowering users to access relevant information and engage in dynamic dialogues [17]. Building on previous studies and feedback from interventionists, young survivors of breast cancer, and health care professionals, we developed an intelligent interactive mobile app called “*AI-TA*” (a WeChat Mini Program) guided by a person-centered care (PCC) framework [12,18,19]. The PCC emphasizes collaborative partnerships between patients and health care providers [20]. It has been shown to enhance patients’ convictions to engage in desired activities and take responsibility for disease management and clinical outcomes [21-23]. Informed by our pilot study results, we have made necessary adjustments to the intervention strategy and module design [24]. In this study, we expand the sample size to further enhance the effectiveness and generalizability of the findings, hypothesizing that users of *AI-TA* will experience significant improvements in psychological symptoms.

### Objective

The purpose of this study is to comprehensively assess the impact of an innovative mobile app, “AI-TA,” which features intelligent design elements, on the psychological health and ongoing symptoms experienced by young survivors of breast cancer.

## Methods

### Study Design

This study was designed as a multicenter, 3-month, parallel group, single-blind, 2-arm randomized controlled trial conducted in 3 university-affiliated hospitals from January 2022 to December 2022. This study investigated the effectiveness of “*AI-TA*” on psychological and related symptoms of young survivors of breast cancer from baseline (T0) to 2 follow-up points (1 month [T1] and 3 months [T2]; Multimedia Appendix 1). The trial was approved by the Chinese Clinical Trial Registry (ChiCTR2200058823).

### Recruitment

Participants were recruited through convenience sampling, aligning with findings from prior studies [25-27], and considering the menopausal age of women. The inclusion and exclusion criteria for the participants are provided below in Textbox 1.

**Inclusion and exclusion criteria.**

**Inclusion criteria**

- Chinese females
- Aged 18-45 years
- Diagnosed with stage 1-3 breast cancer
- Able to access the internet using computer or mobile devices
- Able to read and write in Chinese (traditional or simplified)
- Provided informed consent

**Exclusion criteria**

- Chronic or acute physical conditions that significantly impair daily functioning or require intensive medical care and supervision that could detract from intervention participation or measurement of outcomes
- Serious cognitive or communication barriers (including but not limited to medical diagnoses of advanced dementia, severe aphasia, or other neurological conditions significantly impairing understanding or expression)
- Recurrent or metastatic breast cancer
- Concurrent involvement in other studies

### Randomization

To ensure the quality of the entire study and prevent selection and information bias, our researchers received unified training and were divided into different group roles: (1) recruiter: 2 breast clinical nurses will strictly recruit young survivors of breast cancer according to the inclusion and exclusion criteria and record recruitment information; (2) an independent master candidate randomly assigned participants to 2-armed parallel groups at a 1:1 ratio via a computer-generated digital sequence; (3) intervener: an experienced researcher conducted the interventions, with another researcher serving as the intervention companion, who was responsible for supervision and evaluation; and (4) data collectors: collect and analyze all participants’ data and the results of the intervention by double checking. During the process, blinding was applied to the recruiters and data collectors.

### Procedure

The participants in the control and intervention groups received oral and written instructions on how to use the *AI-TA*, which combines a mobile app with fortnightly web-based follow-up. Each participant used her own WeChat ID to register and log in with a unique or random number for access to *AI-TA* and was told not to discuss the research with other patients so that no identifying information would be linked to them and to reduce contamination. At first enrollment, they were required to complete and return electronic questionnaires, which included sociodemographics, cancer-related characteristics, and psychological and accompanying symptoms in *AI-TA* after informed consent was obtained. The initial baseline evaluation of symptoms was classified at T0. Data collection and assessments of outcomes took place over 2 time points in the follow-up period. T1 assessment took place at 1 month after allocation (intermediate period of intervention), and T2 assessment took place at 3 months after allocation (end point of the intervention). Follow-up assessments were collected via *AI-TA*–assisted self-reported surveys. Each result was saved and available for participants to view their own result at any time. In addition, all participants were awarded CNY 100 (US $13.80) upon completing all the assessments. In addition, there was a questionnaire to assess participants’ interaction of and satisfaction with the *AI-TA* mobile app program for further improvements in the following research (Multimedia Appendix 2).

### Intervention

**Figure 1:**
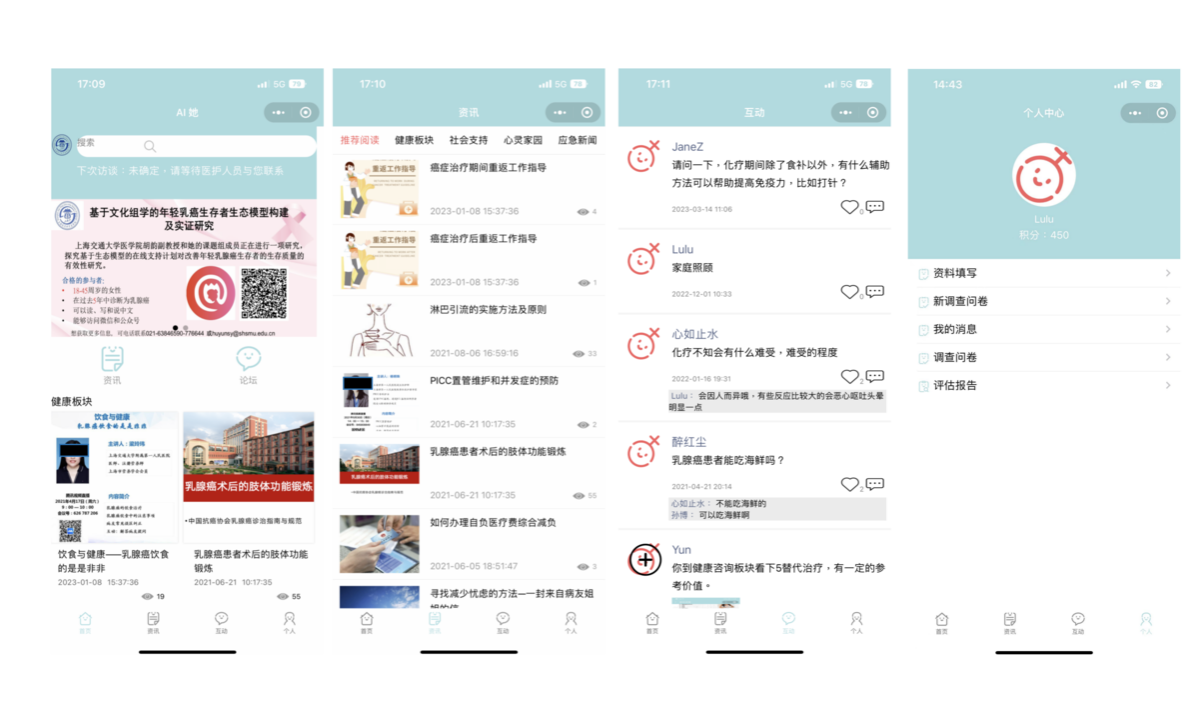
Visual representations of AI-TA.

**Figure 2:**
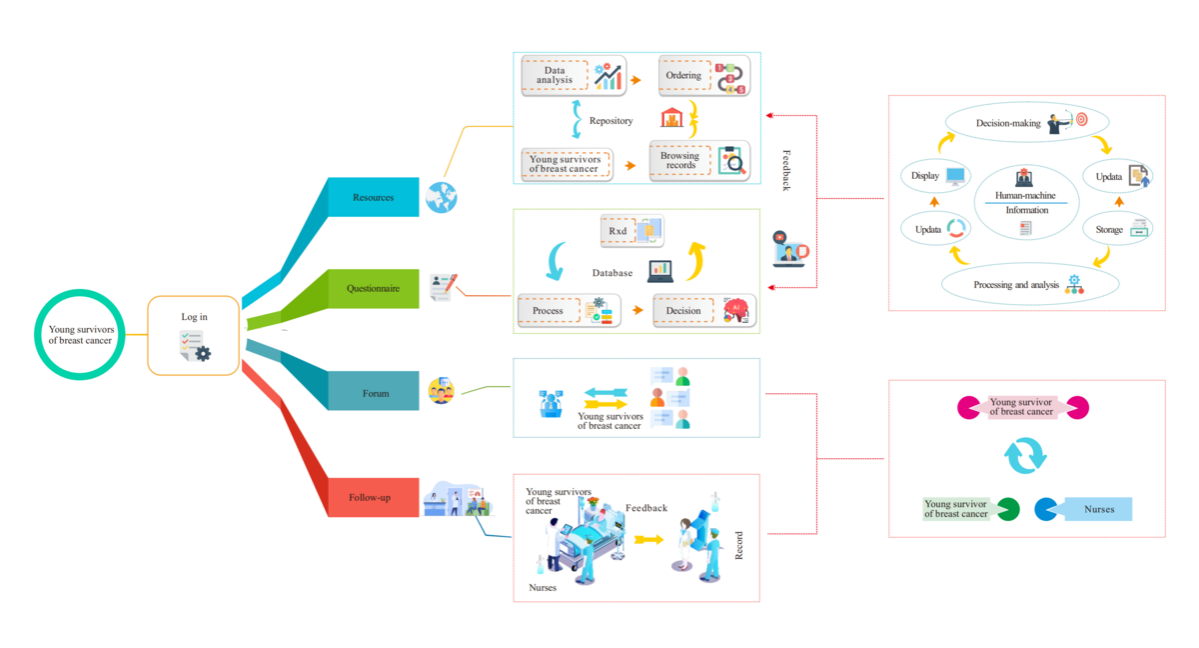
Frame diagram for the intervention design.

*AI-TA* consisted of several modules designed to support young survivors of breast cancer in various aspects of their survivorship. The mobile app stored reliable resources uploaded by health care professionals occasionally from time to time and covered psychological counseling, coping effectiveness, symptom management, social security, etc in text, image, and animation formats. It also allowed participants to synchronously save their log-in, comments, likes, history, duration, and traces. In addition, the health care professionals invited breast clinical experts to hold salon lectures, focusing on common problems in treatments involving diet guidance, functional exercise, tube maintenance, and other guidance. Question and answer sessions were incorporated, and recorded videos were made available for review. Visual representations of *AI-TA* are shown in Figure 1. Additional details relating to the intervention construction can be found in Figure 2.

### AI-Driven System

An AI-driven system is central to our intervention, using text extraction techniques and behavioral data analysis to provide personalized recommendations. This innovative approach significantly enhances user engagement and retention by tailoring content to individual preferences and interests. The following functions were undertaken: (1) personalized content delivery (the AI system analyzes responses from questionnaires to prioritize issues and deliver tailored content; for instance, if young survivors of breast cancer reported low physical activity and poor sleep quality, keywords such as “physical,” “exercise,” “activity,” and “sleep” were used to recommend relevant articles), (2) symptom tracking and management (the system regularly tracks survivors’ symptoms and allows health care professionals to update and tailor content based on their browsing preferences, enhancing the relevance and effectiveness of the information provided), (3) data monitoring and evaluation (AI algorithms can calculate and monitor assessment progress as well as synchronous storage in the server backend), (4) social support network (AI can enable the formation of connections in forums with independent YBCS; it fosters a supportive web-based environment through interactive question and answer sessions, experience sharing, and emotional support), and (5) privacy and security (adhering to health care data regulations, the AI system uses encryption measures to ensure the utmost privacy and security of user data; AI-driven system is a vital link that enables the exchange and feedback of information in peer-to-peer interactions between young survivors of breast cancer and health care professionals and enhances the interactive experience). This dynamic approach ensures that the intervention remains relevant, engaging, and supportive of the unique needs of survivors.

### 2-Way Web-Based Follow-Up

Furthermore, the intervention program included fortnightly web-based follow-up using 2-way communication through private messages or calls. This approach encouraged narration from young survivors of breast cancer and aimed to establish a partnership using PCC communication skills such as open-ended questions, reflections, and summaries. In the initial conversation, health care professionals focused on listening to survivors’ narratives about daily life events and customs (diet, motion, pressure, hobbies, relationships, and sharing) to build trust relationships. The subsequent step entailed the anticipation and cocreation of a health plan jointly based on their feedback through discussion and agreement, including goals, resources, and needs. The contents regarding what participants had talked about, how they felt, what goals they had, and what they wanted to accomplish will be the points for the forthcoming conversations to consider. During the 3-month intervention, participants were also free to get in touch with the health care professionals during office hours. Each follow-up was recorded and uploaded to the platform.

### General Information Support

The control group was granted access to general information on the mobile app, with all modules available in *AI-TA* except for forums and intelligent recommendations. This meant that they could not participate in the forum and obtain recommendations provided by the system based on questionnaire results. In addition, young survivors of breast cancer in the control group also had no follow-up conversations.

### Measures and Instruments

A comprehensive set of questions was used to assess participants’ sociodemographic and health characteristics, including age, height, weight, habitation, educational attainment, marital status, employment, income, offspring, parent, cancer stage (stages 1-3), cancer type, diagnosis time, and treatment.

#### Primary Outcome Measures

##### Psychological Distress and Frequency

The Memorial Symptom Assessment Scale-Short Form (MSAS-SF) was used to assess the frequency and severity of psychological symptoms during the past 7 days [28]. The distress level of each symptom was rated on a 5-point Likert scale (0=“not at all,” 1=“a little bit,” 2=“somewhat,” 3=“quite a bit,” and 4=“very much”). If the symptom was not present, a value of 0 was assigned. The frequency of psychological symptoms is rated from 1 to 4 (1=“rarely” to 4=“almost constantly”).

##### Self-Efficacy

The Cancer Behavior Inventory-Brief Version (CBI-B) developed by Heitzmann et al [29] was adopted to rate self-efficacy for coping with cancer. It is used to assess four factors: (1) maintaining independence and positive attitude, (2) participating in medical care, (3) coping and stress management, and (4) managing affect. There are 12 items in total (rated on a 9-point Likert scale, ranging from 1=“not at all confident” to 9=“totally confident”); a score≤36 is considered low, a score between 37 and 72 is considered moderate, and a score between 73 and 108 is considered high.

##### Social Support

The Social Support Rating Scale (SSRS) is a 10-item questionnaire developed by Xiao [30] for measuring social support, including objective social support, subjective social support, and use of social support. A higher score indicated more social support. The SSRS has been widely used and has shown acceptable reliability and validity in the cancer population. An SSRS score≤22 is considered poor social support, a score between 23 and 44 is considered moderate social support, and a score between 45 and 66 indicates adequate social support.

#### Secondary Outcome Measures

The Functional Assessment of Cancer Therapy-Breast (FACT-B) translated and adapted by Wan et al [31] was used to evaluate the quality of life of patients with breast cancer. The 5 dimensions included physical well-being, social or family well-being, emotional well-being, functional well-being, and additional concerns (cancer type–specific questions). A total of 36 items were scored on a 5-point Likert scale (0=“not at all,” 1=“a little bit,” 2=“somewhat,” 3=“quite a bit,” and 4=“very much”). Among them, 19 items were scored in a reverse manner. Higher scores represent better quality of life.

### Sample Size

This study used G*Power (version 3.1; HHU) to calculate the necessary sample size. On the basis of a similarly designed study, mobile app support reduced the psychological symptoms among survivors of breast cancer with an effect size of 0.77 [32]. Considering the conservative estimate and the variability of previous pilot research [24] as well as the statistical power [33], we estimated that 66 participants were needed to compare between-group differences and present a large effect size (*d*=0.8) in the primary outcome after intervention, with an α level of .05 (2-sided test), 80% statistical power, 1:1 allocation rate, and 20% attrition rate. Thus, a final sample of 124, with 62 (50%) individuals in each group, was adequate.

### Ethical Considerations

The trial complied with the ethical guidelines of the Declaration of Helsinki and was approved by the ethics committee of Public Health and Nursing Research, School of Medicine, Shanghai Jiao Tong University (SJTUPN-201803). All participants provided electronic informed consent before enrollment in the study. All data and information were anonymized according to the established guidelines, and a password-protected document containing participants’ personal information was stored on secure servers.

### Statistical Analysis

Data analysis was performed using SPSS (version 26.0; IBM Corp). Descriptive and comparative statistics were used to characterize the study groups (eg, percentage or mean and SD). A total of 2-sample *t* tests (2-tailed) and chi-square or Fisher exact tests were used where appropriate, and these tests assessed demographic variable differences between the intervention and control groups. Before performing the *t* test, the continuous variables were checked for normality using the Shapiro-Wilk test, and all the data were revealed to be normal (*P*>.05). To confirm the improvements in psychological symptoms, the baseline and postintervention results of the dependent variables were analyzed using the paired *t* tests, whereas the 2-sample *t* tests were used to detect differences between the intervention and control groups at each time point. To estimate the effects of the intervention on the outcomes over time, a linear mixed effect model for repeated measurements was performed. The main effects of group, time, and group×time interaction effects were examined. The significance level was set at *P*<.05 (2 sided).

For this study, both intention-to-treat (ITT) and per-protocol (PP) analyses were conducted. The primary analysis used an ITT approach, which can reflect the results of all participants randomly assigned to receive intervention; missing fields were imputed with the expectation-maximization algorithm. Post hoc sensitivity analyses for missing data were performed to ensure the integrity and reliability of the trial outcomes (Multimedia Appendix 3 [24]). The PP group analysis included participants who fully followed the intervention protocol. The primary end points for evaluating the efficacy of *AI-TA* were MSAS-SF, CBI-B, and SSRS to assess psychological symptoms (distress and frequency), self-efficacy, and social support, respectively, at T2. Secondary end point was FACT-B measures of quality of life.

## Results

### Participants

**Figure 3:**
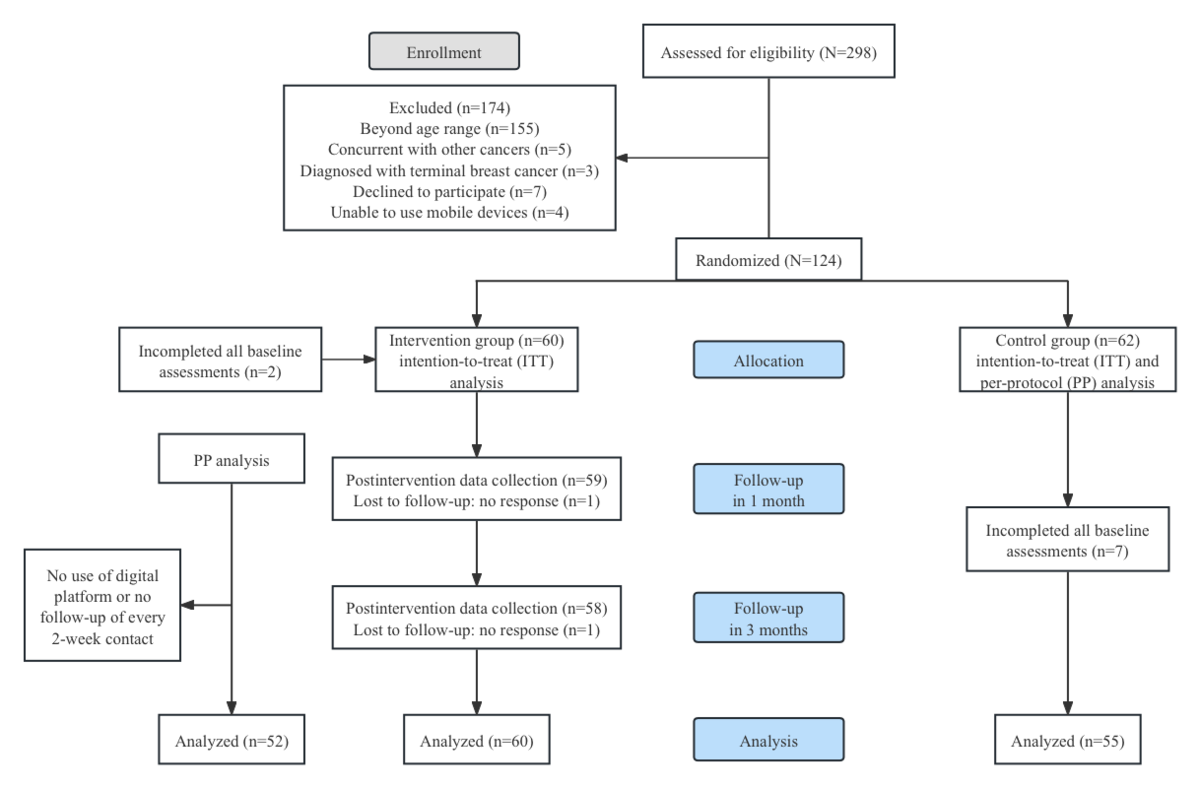
A flowchart of the study participants.

Data were collected through questionnaires at T0, T1, and T2. Approximately 7.3% (9/124) of participants (2/62, 3% in the intervention group and 7/62, 11% in the control group) did not complete all baseline assessment at T0. At T1 and T2, 1.7% (2/115) of participants did not return their questionnaires despite being reminded. A flowchart of the study participants is given in Figure 3.

### Overview

Table 1 provides an overview of the sociodemographic and health characteristics of young survivors of breast cancer at T0. In this study, the participants had an average age of 40.21 (SD 4.24) years, a mean height of 161.16 (SD 4.24) cm, and a mean weight of 55.9 (SD 8.56) kg. The average time since diagnosis was 1.42 (SD 0.3) years. Approximately 89.6% (103/115) participants were living in urban areas and about 90.4% (104/115) were married. Approximately 93.9% (108/115) had children, and 81.7% (94/115) of the participants’ parents were in good health. A total of >50% were employed, and their monthly income was >CNY 10,000 (US $1379.44). In addition, participants tended to be highly educated; approximately 50.4% (58/115) were college graduates and some had graduate degrees. Almost 94.8% (109/115) had invasive breast cancer, and approximately 65.2% (75/115) were diagnosed with stage 2 or stage 3 breast cancer. A total of 11.3% (13/115) only underwent surgical treatment, 42.6% (49/115) only received adjuvant treatment, and 46.1% (53/115) had both. No significant differences were observed in the demographic characteristics between the intervention and control groups, except that the time since diagnosis of participants in the control group was significantly longer than that in the intervention group (*P*=.03). However, this difference did not remain when comparing the control and PP groups (*P*=.18).

**Table 1:** Participant characteristics at baseline (N=115).

Characteristics		Control group (n=55)	ITT^a^ group (n=60)	ITT group, *P* value		PP^b^ group (n=52)		PP group, *P* value	
Age (y), mean (SD)		40.02 (4.32)	40.40 (4.16)	.68		40.66 (4.34)		.27	
Height (cm), mean (SD)		160.55 (4.36)	161.76 (4.12)	.19		161.71 (4.43)		.32	
Weight (kg), mean (SD)		55.37 (7.51)	56.43 (9.61)	.57		56.60 (9.56)		.86	
Diagnosis (y), mean (SD)		1.53 (0.34)	1.31 (0.26)	.03		1.50 (0.29)		.18	
**Residence, n (%)**					.62				.69
	Urban	48 (87.3)	55 (91.7)			50 (96.2)			
	Rural	7 (12.7)	5 (8.3)			2 (3.8)			
**Marital status, n (%)**					.74				.09
	Married	51 (92.7)	53 (88.3)			48 (92.4)			
	Single	3 (5.5)	5 (8.3)			2 (3.8)			
	Other	1 (1.8)	2 (3.4)			2 (3.8)			
**Have a child, n (%)**					.62				.63
	Yes	52 (94.5)	56 (93.3)			49 (94.2)			
	No	3 (5.5)	4 (6.7)			3 (5.8)			
**Parent, n (%)**					.99				.44
	Both	46 (83.6)	48 (80)			44 (84.5)			
	Either	7 (12.7)	11 (18.3)			8 (15.5)			
	Neither	2 (3.7)	1 (1.7)			0 (0)			
**Work status, n (%)**					.95				.55
	Unemployed	19 (34.5)	18 (30)			14 (26.9)			
	Employed	36 (63.5)	42 (70)			38 (73.1)			
**Education level, n (%)**					.60				.36
	Less than or equal to junior college	24 (43.6)	33 (55)			26 (50)			
	College	26 (47.3)	23 (38.3)			23 (44.2)			
	Postgraduate	5 (9.1)	4 (6.7)			3 (5.8)			
**Monthly income (CNY ¥), n (%)**					.57				.90
	<5000 (US $689.72)	11 (20)	11 (18.3)			8 (15.5)			
	5000-10,000 (US $689.72-1379.44)	15 (27.3)	17 (28.3)			15 (28.8)			
	>10,000 (US $1379.44)	29 (52.7)	32 (53.4)			29 (55.7)			
**Type of breast cancer, n (%)**					.45				.89
	Invasive	51 (92.7)	58 (96.7)			50 (96.2)			
	Noninvasive	4 (7.3)	2 (3.3)			2 (3.8)			
**Stage of breast cancer, n (%)**					.13				.07
	1	19 (34.5)	21 (35)			14 (26.9)			
	2	30 (54.5)	28 (46.7)			28 (53.8)			
	3	6 (11)	11 (8.3)			10 (19.3)			
**Therapy for breast cancer, n (%)**					.34				.66
	Operation	5 (9.1)	8 (13.3)			3 (5.8)			
	Adjuvant therapy^c^	26 (47.3)	23 (38.4)			20 (38.5)			
	Both	24 (43.6)	29 (48.3)			29 (55.7)			

^a^ITT: intention-to-treat.

^b^PP: per-protocol.

^c^Includes radiotherapy, chemotherapy, endocrine therapy, etc.

### The Effects on Primary Outcomes

Table 2 presents the overall test results of the intervention effect on psychological symptoms through repeated measures analysis. There were statistically significant group effects, time effects, and group×time interaction effects on the changes in the MSAS-SF and CBI-B scores. Significant time effects (*F*_2,108_=236.123; *P*<.001) and group×time interaction effects (*F*_2,108_=36.639; *P*<.001) were found in the ITT analysis for the SSRS score, but only a significant time effect (*F*_1.767,95.423_=231.187; *P*<.001) was found in the PP analysis.

As demonstrated in Table 3, there were no significant differences in any of the variables between groups at T0, which supported successful randomization. At T2, psychological distress (ITT vs PP vs control: 0.40 vs 0.41 vs 0.93; *P*<.001) was significantly different among the groups. In addition, each group exhibited significant differences (ITT vs PP vs control: 92.83 vs 89.30 vs 85.65; *P*<.001) in self-efficacy; however, compared with those in the control group, the ITT group (*P*=.002) and PP group (*P*<.001) did not show significant increases in social support scores until T2.

**Table 2:** Repeated measures analysis of variance results for total scores.

Variable	ITT^a^				PP^b^		
	Group effect, *F* value (*P* value)	Time effect, *F* value (*P* value)	Group×time effect, *F* value (*P* value)	Group effect, *F* value (*P* value)		Time effect, *F* value (*P* value)	Group×time effect, *F* value (*P* value)
MSAS-SF^c^	14.118 (<.001)	75.718 (<.001)	2.219 (<.001)	13.916 (<.001)		75.007 (<.001)	2.222 (<.001)
CBI-B^d^	7.956 (.007)	526.864 (<.001)	23.850 (<.001)	7.838 (.007)		529.502 (<.001)	23.558 (<.001)
SSRS^e^	0.099 (.75)	236.123 (<.001)	36.639 (<.001)	0.123 (.19)		231.187 (<.001)	37.928 (.16)
FACT-B^f^	6.081 (.02)	275.261 (<.001)	36.978 (<.001)	2.571 (.02)		325.216 (<.001)	45.457 (<.001)

^a^ITT: intention-to-treat; n=60 in the intervention group and n=55 in the control group.

^b^PP: per-protocol; n=52 in the intervention group and n=55 in the control group.

^c^MSAS-SF: Memorial Symptom Assessment Scale-Short Form.

^d^CBI-B: Cancer Behavior Inventory-Brief Version.

^e^SSRS: Social Support Rating Scale.

^f^FACT-B: Functional Assessment of Cancer Therapy-Breast.

**Table 3:** Changes in psychological and related symptoms over time between each group.

Variable		Control group (n=55), mean (SD)	ITT^a^ group (n=60)		PP^b^ group (n=52)	
			Values, mean (SD)	*P* value^c^	Values, mean (SD)	*P* value^c^
**MSAS-SF^d^**						
	T0	1.51 (0.50)	1.56 (0.47)	.25	1.59 (0.43)	.20
	T1	1.23 (0.37)^e^	1.17 (0.46)^e^	<.001	1.07 (0.50)^e^	.37
	T2	0.91 (0.22)^e^	0.68 (0.21)^e,f^	<.001	0.88 (0.20)^e^	.05
**Psychological frequency**						
	T0	1.52 (0.29)	1.43 (0.36)	.67	1.50 (0.25)	.49
	T1	1.45 (0.14)	1.41 (0.19)	.006	1.36 (0.17)	<.001
	T2	0.95 (0.21)^e,f^	0.83 (0.13)	<.001	0.77 (0.20)^e,f^	<.001
**Psychological distress**						
	T0	1.59 (0.14)	1.48 (0.11)	.38	1.37 (0.18)	.75
	T1	1.22 (0.12)^e^	0.79 (0.25)^e^	<.001	0.80 (0.13)^e^	<.001
	T2	0.93 (0.17)^e,f^	0.40 (0.16)^e,f^	<.001	0.41 (0.19)^e,f^	<.001
**Global distress**						
	T0	1.46 (0.26)	1.30 (0.17)	.07	1.31 (0.22)	.92
	T1	1.23 (0.11)^e^	0. 94 (0.23)^e^	<.001	0.95 (0.14)^e^	<.001
	T2	1.03 (0.13)^e,f^	0.41 (0.20)^e,f^	<.001	0.54 (0.11)^e,f^	<.001
**Physical distress**						
	T0	1.08 (0.15)	1.16 (0.10)	.30	1.13 (0.17)	.41
	T1	0.75 (0.14)^e^	0.56 (0.25)^e^	.002	0.61 (0.15)^e^	.03
	T2	0.49 (0.15)^e,f^	0.32 (0.12)^e,f^	<.001	0.39 (0.11)^e,f^	.01
**CBI-B^g^**						
	T0	37.07 (3.50)	36.13 (6.72)	.08	36.62 (3.81)	.43
	T1	65.35 (3.06)^e^	82.83 (5.70)^e^	<.001	77.12 (4.27)^e^	<.001
	T2	85.65 (2.79)^e,f^	92.83 (3.04)^e,f^	<.001	89.30 (5.18)^e,f^	<.001
**Maintaining independence and a positive attitude**						
	T0	5.27 (1.56)	5.39 (1.88)	.79	5.28 (1.63)	.22
	T1	6.00 (1.41)^e^	6.98 (1.88)^e^	.04	6.97 (1.88)^e^	.02
	T2	6.48 (1.35)^e,f^	7.84 (1.48)^e,f^	.001	7.85 (1.47)^e,f^	.001
**Participating in medical care**						
	T0	6.32 (1.55)	6.27 (2.06)	.57	6.07 (1.74)	.55
	T1	6.89 (1.31)^e^	7.28 (1.56)^e^	.01	7.27 (1.56)^e^	.03
	T2	7.77 (1.19)^e,f^	7.78 (1.63)^e^	.03	7.80 (1.35)^e,f^	.05
**Coping and stress management**						
	T0	4.91 (1.67)	5.07 (1.69)	.91	5.14 (1.16)	.89
	T1	5.76 (1.24)^e^	6.91 (1.55)^e^	.001	6.88 (1.56)^e^	.04
	T2	6.79 (1.18)^e,f^	7.74 (1.27)^e,f^	<.001	7.70 (1.26)^e,f^	<.001
**Managing affect**						
	T0	5.55 (0.95)	5.40 (1.81)	.14	5.39 (1.85)	.13
	T1	5.60 (1.67)^e^	6.62 (1.57)^e^	<.001	6.62 (1.58)^e^	<.001
	T2	6.76 (0.59)^e,f^	7.62 (1.47)^e,f^	<.001	7.63 (1.45)^e,f^	<.001
**SSRS^h^**						
	T0	39.33 (7.39)	38.04 (8.19)	.07	39.47 (7.24)	.50
	T1	42.31 (6.68)^e^	42.67 (7.94)^e^	.73	42.45 (7.80)^e^	.74
	T2	45.10 (6.44)^e,f^	50.09 (4.95)^e,f^	.002	49.78 (5.09)^e,f^	<.001
**Objective social support**						
	T0	10.24 (3.58)	9.53 (3.08)	.26	9.70 (3.12)	.53
	T1	11.62 (3.24)^e^	11.76 (3.51)^e^	.62	11.82 (3.32)^e^	.78
	T2	12.69 (3.19)^e,f^	14.66 (2.63)^e,f^	.02	14.88 (2.57)^e,f^	.01
**Subjective social support**						
	T0	21.62 (4.19)	21.72 (4.99)	.36	21.33 (4.66)	.42
	T1	22.48 (3.87)^e^	22.62 (4.88)^e^	.80	23.52 (4.42)^e^	.61
	T2	23.4 (3.71)^e,f^	25.28 (2.74)^e,f^	.01	25.61 (2.62)^e,f^	.03
**Use of social support**						
	T0	7.48 (2.03)	7.57 (2.03)	.31	7.36 (2.04)	.13
	T1	8.21 (1.8)^e^	8.29 (2.22)^e^	.40	8.39 (2.21)^e^	.24
	T2	9 (1.75)^e,f^	10.16 (1.33)^e,f^	.007	10.09 (1.38)^e,f^	.04

^a^ITT: intention-to-treat.

^b^PP: per-protocol.

^c^*P* value of between-group differences.

^d^MSAS-SF: Memorial Symptom Assessment Scale-Short Form.

^e^Compared with T0, *P*<.05.

^f^Compared with T1, *P*<.05.

^g^CBI-B: Cancer Behavior Inventory-Brief Version.

^h^SSRS: Social Support Rating Scale.

### The Effects on Secondary Outcomes

First, the analysis highlighted a statistically significant group effect (*P*=.02), time effect (*P*<.001), and group×time interaction effect (*P*<.001) for quality of life in both the ITT and PP groups (Table 2).

The results at T1 (ITT vs PP vs control: 106.68 vs 105.73 vs 100.33; *P*<.05) and T2 (ITT vs PP vs control: 124.47 vs 126.04 vs 113.50; *P*<.001) indicated that there was significant improvement in overall quality of life. Notably, significant differences between and within all groups were found in functional well-being and additional concerns both in T1 and T2. Compared with those in the control group, the ITT and PP groups did not show significant increases in physical, social or family, and emotional well-being until T2 (*P*<.05; Table 4).

**Table 4:** Changes in quality of life over time between each group.

Variable		Control group (n=55), mean (SD)	ITT^a^ group (n=60)			PP^b^ group (n=52)	
			Values, mean (SD)	*P* value^c^	Values, mean (SD)		*P* value^c^
**FACT-B** ^d^							
	T0	92.48 (15.15)	90.02 (12.44)	.52	91.64 (12.85)		.30
	T1	100.33 (12.32)^e^	106.68 (12.49)^e^	.01	105.73 (12.54)^e^		.02
	T2	113.50 (11.20)^e,f^	124.47 (9.14)^e,f^	<.001	126.04 (10.69)^e,f^		<.001
**Physical well-being**							
	T0	20.5 (4.27)	20.04 (5.05)	.65	19.73 (4.99)		.75
	T1	21.17 (3.66)^e^	21.61 (4.44)^e^	.45	22.8 (4.41)^e^		.44
	T2	22 (3.57)^e,f^	24.91 (3.00)^e,f^	<.001	24.48 (3.73)^e,f^		<.001
**Social or family well-being**							
	T0	19.86 (5.07)	20.11 (7.93)	.09	19.48 (5.35)		.07
	T1	20.52 (3.76)^e^	21.31 (4.37)^e^	.39	21.10 (4.51)^e^		.39
	T2	21.45 (3.47)^e,f^	24.89 (2.88)^e,f^	<.001	24.73 (2.24)^e,f^		<.001
**Emotional well-being**							
	T0	12.88 (4.27)	12.87 (4.28)	.98	12.48 (4.51)		.84
	T1	15.45 (3.01)^e^	15.98 (2.57)^e^	.39	18.36 (4.78)^e^		.40
	T2	17.24 (2.99)^e,f^	18.50 (2.57)^e,f^	<.001	22.48 (3.51)^e,f^		.02
**Functional well-being**							
	T0	12.57 (5.11)	12.89 (6.75)	.81	12.14 (5.06)		.48
	T1	15.55 (3.92)^e^	17.86 (4.72)^e^	.008	17.91 (4.21)^e^		.008
	T2	18.29 (3.63)^e,f^	23.85 (3.84)^e,f^	<.001	22.14 (3.08)^e,f^		<.001
**Additional concerns (cancer type**–**specific questions)**							
	T0	26.67 (3.69)	26.91 (5.85)	.57	26.48 (4.11)		.48
	T1	27.64 (3.35)^e^	29.92 (4.78)^e^	.02	28.00 (3.66)^e^		.02
	T2	28.52 (3.38)^e,f^	32.31 (3.29)^e,f^	<.001	31.17 (3.52)^e,f^		<.001

^a^ITT: intention-to-treat.

^b^PP: per-protocol.

^c^*P* value of between-group differences.

^d^FACT-B: Functional Assessment of Cancer Therapy-Breast.

^e^Compared with T0, *P*<.05.

^f^Compared with T1, *P*<.05.

## Discussion

### Principal Findings

This randomized controlled trial examined the effectiveness of an internet-enabled, mobile, intelligent interactive intervention for young survivors of breast cancer over a 3-month period. The findings demonstrated the benefits of using a mobile app and engaging in 2-way web-based follow-up. Significant improvements were observed in psychological symptoms, including distress and frequency, indicating the positive impact of the intervention. In addition, there was a noticeable trend toward improvement in quality-of-life outcomes, with both the ITT and PP analyses showing consistent overall outcomes.

We observed that the MSAS-SF score decreased from moderate to mild in all groups, and psychological distress also significantly decreased by 1.08 in the ITT group and 0.96 in the PP group from baseline; these findings were more pronounced than those of American survivors of breast cancer [32] and survivors of lung cancer [34]. In addition, a significant reduction between groups in the frequency of psychological problems was found (eg, sadness, worry, irritability, and nervousness), with 0.83 in the ITT group and 0.77 in the PP group at T2. Clinical evidence has confirmed that survivors of cancer who approach people considered isolated and marginalized with stigmatized conditions and underserved populations to confide negative emotions and relieve themselves may become stuck in a psychological trap [35]. *AI-TA* adopted multiple approaches to alleviate psychological distress and reduce the frequency of psychological symptoms. For example, individuals could connect and offer spiritual support to each other in a private forum, allowing young survivors of breast cancer to openly share experience and advice, thereby overcoming the hesitation often caused by traditional cultural norms. Web-based follow-up enables continuous care between health care professionals and survivors, providing targeted educational contents such as information about stress relief, emotional management, and other relevant information [36]. By integrating the assessments from AI-driven system with qualitative insights from interviews, the follow-up can be more accurate to address the nuanced needs of young survivors of breast cancer, ensuring that the care provided is both relevant and effective. In this study, young survivors of breast cancer had a lower frequency of psychological symptoms at baseline and therefore had little margin for improvement at T1 and T2 in the ITT group; conversely, the PP group demonstrated notable enhancements. This disparity may relate to the duration or intensity of the intervention [37]. In addition, the greater increase in physical and global distress in the intervention group identified the necessity of intelligent interactive support for young survivors of breast cancer.

The findings reported in this study align with the literature, indicating that the intervention program *AI-TA* had a positive effect on increasing self-efficacy levels, as reported previously [32]. In particular, the ITT and PP groups had already reached a high level of self-efficacy at T1, which was faster than the control group. Among them, coping and stress management showed a borderline significant trend between groups at T1, and it became better at T2. This is likely because these survivors lacked motivation and familiarity with *AI-TA* at first, resulting in insufficient in-depth effects on young survivors of breast cancer [38]. Although the intervention group exhibited more favorable changes in several self-efficacy variables, all the groups experienced positive changes, which indicated that the general information support also played a certain role in promoting young survivors of breast cancer.

In this study, the changes in symptoms among survivors of breast cancer were similar to changes in their levels of self-efficacy. Several explanations have been proposed to account for the relationship between syndrome and self-efficacy: patients with cancer with high self-efficacy have a high level of health beliefs, which may promote the recovery from symptoms; in contrast, those with low self-efficacy are prone to negative emotions such as anxiety and depression, which are not conducive to recovery from psychological and physical symptoms [39,40]. AI-driven system and fortnightly web-based follow-up encouraged young survivors of breast cancer to actively engage with the provided content, enhancing their participation and initiative. This interactive process improved their confidence and self-efficacy in managing their symptoms, as the AI continually adapts to their evolving needs and responses.

Furthermore, while all groups’ total social support scores were sustained and reached adequacy by T2, no significant effect was observed across any dimension at T1. This initial absence can be attributed to the challenges faced by young survivors of breast cancer. Frequently undergoing treatments and grappling with severe side effects, young survivors likely found themselves with limited energy to engage with the AI-driven tool (*AI-TA*) or to communicate effectively with health care professionals [41]. Besides, research has identified several factors that affect the perception of social support of survivors of cancer. Specifically, young patients with a collectivist orientation who value in-group solidarity and interdependence may feel alienated from or resist joining groups perceived as outside their usual social circles in a short term [42]. Initially, *AI-TA* may not show a significant impact. The novelty of such apps and their integration into survivors’ lives requires time to manifest tangible benefits. However, as these AI-driven systems evolve to more accurately assess and respond to daily symptoms, their potential to significantly enhance health management and symptom control for young survivors with breast cancer grows. Over time, continued engagement with *AI-TA* is likely to foster social support, deeper understanding of the disease, and overall better well-being for them. The gradual accumulation of these positive effects underscores the promise of long-term interventions to bolster survivors’ outcomes.

This work revealed that the *AI-TA* mobile app has been shown to work effectively in improving quality of life. The findings in physical, social or family, and emotional well-being of these survivors did not increase until T2. Previous literature has also been published in the field of internet-based or computer-based interventions for survivors of breast cancer, and the results indicate that internet support has no significant impact on quality of life in recently diagnosed survivors of breast cancer [43]. Particularly in the early postoperative period and before and after chemotherapy, the recovery of physical well-being and role function was slower [44]. In addition to confronting the challenges of disease itself, young survivors of breast cancer often experience negative emotions associated with work, childbirth, support, and other pressures as well as feelings about being abandoned by the medical system [45]. The integration of web-based follow-up through AI-driven system fosters continuous interaction and support, which is crucial for these survivors managing sensitive and often underdiscussed topics such as sexual health. These not only allow real-time monitoring and assistance but also facilitate a space for them to seek guidance and share experiences securely and comfortably.

### Limitations

There are several limitations to this research that weaken the generalizability of these findings and warrant further investigation. Firstly, because of the relatively small population size and heterogeneity of treatment, a small study may not detect significant effects on outcomes related to the whole psychological symptom. Secondly, it is possible that young survivors of breast cancer with different types of cancer would react differently to the content of this intervention. Furthermore, the duration of this study was short; thus, in the future, long-term interventions could be carried out to detect differences between groups.

### Conclusions

The mobile app *AI-TA* demonstrated significant benefits in addressing the psychological health needs of young survivors of breast cancer during their survivorship journey. The consistent duration, intelligent support, and ease of interaction and web-based follow-up facilitated through digital platforms contributed to the success of the intervention. Specifically, AI-driven features such as personalized content delivery based on user feedback, symptom tracking and management, and interactive support networks have proven crucial for enhancing self-efficacy and social support among these survivors. Emphasis should be placed on optimizing the frequency of interaction and content delivery during an intervention to sustain user engagement without inducing fatigue. The observed effect size on psychological and related symptoms warrants further exploration, prompting future research to expand and investigate the efficacy of such AI-driven interventions in larger trials and across diverse populations over extended periods.

## Appendix

Multimedia Appendix 1 CONSORT-EHEALTH (Consolidated Standards of Reporting Trials of Electronic and Mobile Health Applications and Online Telehealth) form V1.6.

Multimedia Appendix 2 Questionnaire to assess participants’ interaction and satisfaction with the AI-TA mobile app program.

Multimedia Appendix 3 Post hoc sensitivity analyses for missing data.

## Abbreviations

AI: artificial intelligence
CBI-B: Cancer Behavior Inventory-Brief Version
FACT-B: Functional Assessment of Cancer Therapy-Breast
ITT: intention-to-treat
MSAS-SF: Memorial Symptom Assessment Scale-Short Form
PCC: person-centered care
PP: per-protocol
SSRS: Social Support Rating Scale

## References

[1] Siegel RL, Miller KD, Wagle NS, Jemal A (2023). Cancer statistics, 2023. CA Cancer J Clin.

[2] Fan L, Strasser-Weippl K, Li JJ, St Louis J, Finkelstein DM, Yu KD, Chen WQ, Shao ZM, Goss PE (2014). Breast cancer in China. Lancet Oncol.

[3] Population factsheets. International Agency for Research on Cancer.

[4] Li T, Mello-Thoms C, Brennan PC (2016). Descriptive epidemiology of breast cancer in China: incidence, mortality, survival and prevalence. Breast Cancer Res Treat.

[5] Cai T, Huang Y, Huang Q, Xia H, Yuan C (2021). Symptom trajectories in patients with breast cancer: an integrative review. Int J Nurs Sci.

[6] Bower JE, Partridge AH, Wolff AC, Thorner ED, Irwin MR, Joffe H, Petersen L, Crespi CM, Ganz PA (2021). Targeting depressive symptoms in younger breast cancer survivors: the pathways to wellness randomized controlled trial of mindfulness meditation and survivorship education. J Clin Oncol.

[7] Nakamura Z, Deal A, Nyrop K, Chen YT, Quillen LJ, Brenizer T, Muss HB (2021). Serial assessment of depression and anxiety by patients and providers in women receiving chemotherapy for early breast cancer. Oncologist.

[8] Miaja M, Platas A, Martinez-Cannon BA (2017). Psychological impact of alterations in sexuality, fertility, and body image in young breast cancer patients and their partners. Rev Invest Clin.

[9] Zhang Y, Zhang X, Li N, He H, Chen J, Zhu M, Zhang M (2023). Factors associated with psychosocial adjustment in newly diagnosed young to middle-aged women with breast cancer: a cross-sectional study. Eur J Oncol Nurs.

[10] Hu Y, Xu J, Wang X, Shi Y, Chen M, Im EO (2021). Socio-ecological environmental characteristics of young Chinese breast cancer survivors. Oncol Nurs Forum.

[11] Schmidt ME, Scherer S, Wiskemann J, Steindorf K (2019). Return to work after breast cancer: the role of treatment-related side effects and potential impact on quality of life. Eur J Cancer Care (Engl).

[12] Jiang L, Wang X, Hu Y (2022). Research of comprehensive needs of young breast cancer survivors based on user portrait. Nurs J Chin People Liberation Army.

[13] Chen SQ, Sun N, Ge W, Su JE, Li QR (2020). The development process of self-acceptance among Chinese women with breast cancer. Jpn J Nurs Sci.

[14] Kane K, Kennedy F, Absolom KL, Harley C, Velikova G (2023). Quality of life support in advanced cancer-web and technological interventions: systematic review and narrative synthesis. BMJ Support Palliat Care.

[15] Sotirova MB, McCaughan EM, Ramsey L, Flannagan C, Kerr DP, O'Connor SR, Blackburn NE, Wilson IM (2021). Acceptability of online exercise-based interventions after breast cancer surgery: systematic review and narrative synthesis. J Cancer Surviv.

[16] Jiang S (2019). Functional interactivity in social media: an examination of Chinese health care organizations' microblog profiles. Health Promot Int.

[17] Weidener L, Fischer M (2024). Role of ethics in developing AI-based applications in medicine: insights from expert interviews and discussion of implications. JMIR AI.

[18] Xie YD, Wang X, Huang Y (2022). A comparative study of medical APP interface design based on emotional design principles. Med Inform.

[19] Hu Y, Cheng C, Chee W, Im EO (2020). Issues in internet-based support for chinese-american breast cancer survivors. Inform Health Soc Care.

[20] Britten N, Ekman I, Naldemirci Ö, Javinger M, Hedman H, Wolf A (2020). Learning from Gothenburg model of person centred healthcare. BMJ.

[21] Pirhonen L, Olofsson EH, Fors A, Ekman I, Bolin K (2017). Effects of person-centred care on health outcomes-a randomized controlled trial in patients with acute coronary syndrome. Health Policy.

[22] Wolf A, Fors A, Ulin K, Thorn J, Swedberg K, Ekman I (2016). An eHealth diary and symptom-tracking tool combined with person-centered care for improving self-efficacy after a diagnosis of acute coronary syndrome: a substudy of a randomized controlled trial. J Med Internet Res.

[23] Fors A, Blanck E, Ali L, Ekberg-Jansson A, Fu M, Lindström Kjellberg I, Mäkitalo Å, Swedberg K, Taft C, Ekman I (2018). Effects of a person-centred telephone-support in patients with chronic obstructive pulmonary disease and/or chronic heart failure - a randomized controlled trial. PLoS One.

[24] Jiang L, Wang X, Xu J, Wu Y, Hu Y (2023). Construction of an intelligent interactive nursing information support system and its application in young breast cancer survivors. Chin J Nurs.

[25] Rosenberg SM, Dominici LS, Gelber S, Poorvu PD, Ruddy KJ, Wong JS, Tamimi RM, Schapira L, Come S, Peppercorn JM, Borges VF, Partridge AH (2020). Association of breast cancer surgery with quality of life and psychosocial well-being in young breast cancer survivors. JAMA Surg.

[26] Kim K, Park H (2021). Factors affecting anxiety and depression in young breast cancer survivors undergoing radiotherapy. Eur J Oncol Nurs.

[27] Xu J, Wang X, Chen M, Shi Y, Hu Y (2021). Family interaction among young Chinese breast cancer survivors. BMC Fam Pract.

[28] Fu L, Hu Y, Lu Z, Zhou Y, Zhang X, Chang VT, Yang Y, Wang Y (2018). Validation of the simplified Chinese version of the memorial symptom assessment scale-short form among cancer patients. J Pain Symptom Manage.

[29] Heitzmann CA, Merluzzi TV, Jean-Pierre P, Roscoe JA, Kirsh KL, Passik SD (2011). Assessing self-efficacy for coping with cancer: development and psychometric analysis of the brief version of the Cancer Behavior Inventory (CBI-B). Psychooncology.

[30] Xiao SY (1994). The theoretical basis and research application of social support rating scale. J Clin Psychiatry.

[31] Wan C, Zhang D, Tang X, Zhang W, Li W, Ren H, He R, Wang W (2002). Introduction on measurement scale of quality of life for patients with breast cancer: Chinese version of FACT-B. China Cancer.

[32] Im EO, Kim S, Yang YL, Chee W (2020). The efficacy of a technology-based information and coaching/support program on pain and symptoms in Asian American survivors of breast cancer. Cancer.

[33] Cohen J (2016). Statistical power analysis. Curr Dir Psychol Sci.

[34] Hung HY, Wu LM, Chen KP (2018). Determinants of quality of life in lung cancer patients. J Nurs Scholarsh.

[35] Wang JH, Adams I, Huang E, Ashing-Giwa K, Gomez SL, Allen L (2012). Physical distress and cancer care experiences among Chinese-American and non-Hispanic White breast cancer survivors. Gynecol Oncol.

[36] Ko NY, Fikre TG, Buck AK, Restrepo E, Warner ET (2023). Breast cancer survivorship experiences among Black women. Cancer.

[37] Tripepi G, Chesnaye NC, Dekker FW, Zoccali C, Jager KJ (2020). Intention to treat and per protocol analysis in clinical trials. Nephrology (Carlton).

[38] Jakob R, Harperink S, Rudolf AM, Fleisch E, Haug S, Mair JL, Salamanca-Sanabria A, Kowatsch T (2022). Factors influencing adherence to mHealth apps for prevention or management of noncommunicable diseases: systematic review. J Med Internet Res.

[39] Yuan L, Yuan L (2021). Effectiveness of nursing Intervention on anxiety, psychology and self-efficacy among elderly patients with acute coronary syndrome after percutaneous coronary intervention: an observational cohort study. Medicine (Baltimore).

[40] Kurt S, Altan Sarikaya N (2022). Correlation of self-efficacy and symptom control in cancer patients. Support Care Cancer.

[41] Zhu J, Ebert L, Liu X, Wei D, Chan SW (2018). Mobile breast cancer e-support program for Chinese women with breast cancer undergoing chemotherapy (part 2): multicenter randomized controlled trial. JMIR Mhealth Uhealth.

[42] To C, Leslie LM, Torelli CJ, Stoner JL (2020). Culture and social hierarchy: collectivism as a driver of the relationship between power and status. Organ Behav Hum Decis Process.

[43] Loiselle CG, Edgar L, Batist G, Lu J, Lauzier S (2010). The impact of a multimedia informational intervention on psychosocial adjustment among individuals with newly diagnosed breast or prostate cancer: a feasibility study. Patient Educ Couns.

[44] Zhou K, Wang W, Zhao W, Li L, Zhang M, Guo P, Zhou C, Li M, An J, Li J, Li X (2020). Benefits of a WeChat-based multimodal nursing program on early rehabilitation in postoperative women with breast cancer: a clinical randomized controlled trial. Int J Nurs Stud.

[45] Matthews H, Semper H (2017). 'Dropped from the system': the experiences and challenges of long-term breast cancer survivors. J Adv Nurs.

