# Supplementary material for: Effects of the “AI-TA” Mobile App With Intelligent Design on Psychological and Related Symptoms of Young Survivors of Breast Cancer: Randomized Controlled Trial
Source: JMIR Mhealth Uhealth. 2024 Jun 4;12:e50783. doi: 10.2196/50783 (PMC11185911; doi:10.2196/50783)
Supplement: Multimedia Appendix 2 [file mhealth_v12i1e50783_app2.docx]

**Multimedia Appendix 1.** Questionnaire to assess participants’interaction of and satisfaction with the“AI-TA”mobile application program.

Questionnaire for User Interaction Satisfaction (Version 7.0)

The QUIS Version 7.0 is arranged in a hierarchical format and contains: (1) an overall reaction to the software, (2) six scales that measure overall reaction ratings of the system: screen factors, terminology and system feedback, learning factors, system capabilities, and optional sections to evaluate specific components of the system. There are 27 items in total (on a 9-point Likert scale; from 0 to 9), a higher score indicates more satisfaction of users.

**Table S1.** User interaction satisfaction (N=115)

| **Variable** | **Mean** | **SD** |
| --- | --- | --- |
| 1. **Overall reaction to the software** | 5.81 | 2.09 |
| 1. **Screen** |  |  |
| 2.1 Reading characters on the screen | 7.24 | 1.61 |
| 2.2 Highlighting simplifies task | 5.99 | 2.43 |
| 2.3 Organization of information | 6.23 | 2.29 |
| 2.4 Sequence of screens | 6.16 | 2.13 |
| 1. **Terminology and system information** |  |  |
| 3.1 Use of terms throughout system | 6.43 | 1.95 |
| 3.2 Terminology related to task | 6.43 | 1.77 |
| 3.3 Position of messages on screen | 6.74 | 1.90 |
| 3.4 Prompts for input | 6.47 | 2.53 |
| 3.5 Computer informs about its progress | 6.40 | 2.32 |
| 3.6 Error messages | 5.97 | 2.57 |
| 1. **Learning** |  |  |
| 4.1 Learning to operate the system | 6.94 | 1.93 |
| 4.2 Exploring new features by trial and error | 6.86 | 1.76 |
| 4.3 Remembering names and use of commands | 6.79 | 1.79 |
| 4.4 Performing tasks is straightforward | 6.53 | 1.97 |
| 4.5 Help messages on the screen | 6.44 | 2.30 |
| 4.6 Supplemental reference materials | 6.53 | 1.99 |
| 1. **System capabilities** |  |  |
| 5.1 System speed | 6.69 | 1.80 |
| 5.2 System reliability | 6.36 | 1.90 |
| 5.3 System tends to be | 6.60 | 2.07 |
| 5.4 Correcting your mistakes | 6.63 | 1.99 |
| 5.5 Designed for all levels of users | 6.30 | 2.07 |
| 1. **Usability and UI** |  |  |
| 6.1 Use of colors and sounds | 6.47 | 1.96 |
| 6.2 System feedback | 6.53 | 2.15 |
| 6.3 System response to errors | 6.36 | 2.15 |
| 6.4 System messages and reports | 6.53 | 2.13 |
| 6.5 System clutter and UI “noise” | 6.40 | 2.31 |
