# Supplementary material for: Effects of the “AI-TA” Mobile App With Intelligent Design on Psychological and Related Symptoms of Young Survivors of Breast Cancer: Randomized Controlled Trial
Source: JMIR Mhealth Uhealth. 2024 Jun 4;12:e50783. doi: 10.2196/50783 (PMC11185911; doi:10.2196/50783)
Supplement: Multimedia Appendix 3 [file mhealth_v12i1e50783_app3.docx]

**Multimedia Appendix 2.** Post hoc sensitivity analyses for missing data

1. Strategic approaches were adopted both before and during the trial regarding avoiding the missing data

1.1 Prior to the trial

Our research team proactively identified potential causes of dropouts or incomplete responses, implementing preemptive strategies such as text message and phone call reminders. A pilot study was undertaken to pinpoint potential shortcomings in data collection methodologies, leading to adjustments in the study design to rectify these issues [1]. Moreover, researchers underwent comprehensive training emphasizing the crucial importance of thorough data collection and the specific procedures designed to minimize data loss. Data collection instruments were crafted to be intuitive, straightforward, and concise, aiming to diminish the chances of partial responses. From the beginning, we made concerted efforts to engage participants, underlining the significance of their ongoing participation and the necessity for full data contribution.

1.2 During the trial

Throughout the trial, we vigilantly monitored the data collection process to swiftly identify and resolve instances of missing data. We established stringent follow-up protocols for participants who were unable to attend visits or complete measurements, employing reminders through phone calls, emails, or postal correspondence. Interim analyses were periodically conducted to gauge the extent and patterns of missing data, enabling us to undertake immediate corrective measures. A responsive approach was maintained towards the needs and concerns of the participants, proactively addressing any factors that could potentially lead to discontinuation or incomplete data submissions.

2. Post hoc sensitivity analyses

Through these comprehensive strategies, the missing rate reduced to 2%, and missing fields were imputed with the Expectation Maximization (EM) Algorithm based on missing completely at random (MCAR) assumptions. Given the potential problems that come with deciding on this imputation method, post hoc sensitivity analyses for missing data were performed to ensure the integrity and reliability of the trial outcomes. The primary endpoints were psychological symptoms, self-efficacy and social support measured by Memorial Symptom Assessment Scale-Short Form (MSAS-SF), Cancer Behavior Inventory-Brief Version (CBI-B) and Social Support Rating Scale (SSRS), respectively at 3 months postintervention. The secondary endpoints were quality of life measured by Functional Assessment of Cancer Therapy-Breast (FACT-B).

**Table1. Pre- and post-Imputation estimates of MSAS-SF and between-group differences at 3 months postintervention.**

| **Number** | **Group** | **Mean(SD)** | ***P* value** |
| --- | --- | --- | --- |
| Original data | ITT | 0.67(0.26) | .035 |
|  | Control | 0.91(0.22) |  |
| 1 | ITT | 0.66(0.21) | <.001 |
|  | Control | 0.91(0.22) |  |
| 2 | ITT | 0.69(0.26) | <.001 |
|  | Control | 0.91(0.22) |  |
| 3 | ITT | 0.68(0.26) | <.001 |
|  | Control | 0.91(0.22) |  |
| 4 | ITT | 0.67(0.27) | <.001 |
|  | Control | 0.91(0.22) |  |
| 5 | ITT | 0.69(0.27) | <.001 |
|  | Control | 0.91(0.22) |  |

**Table2. Pre- and post-Imputation estimates of CBI-B and between-group differences at 3 months postintervention.**

| **Number** | **Group** | **Mean(SD)** | ***P* value** |
| --- | --- | --- | --- |
| Original data | ITT | 90.94(4.87) | <.001 |
|  | Control | 89.30(5.18) |  |
| 1 | ITT | 92.76(3.05) | <.001 |
|  | Control | 89.30(5.18) |  |
| 2 | ITT | 92.63(3.10) | <.001 |
|  | Control | 89.30(5.18) |  |
| 3 | ITT | 92.84(3.04) | <.001 |
|  | Control | 89.30(5.18) |  |
| 4 | ITT | 92.67(3.08) | <.001 |
|  | Control | 89.30(5.18) |  |
| 5 | ITT | 92.68(2.08) | <.001 |
|  | Control | 89.30(5.18) |  |

**Table3. Pre- and post-Imputation estimates of SSRS and between-group differences at 3 months postintervention.**

| **Number** | **Group** | **Mean(SD)** | ***P* value** |
| --- | --- | --- | --- |
| Original data | ITT | 50.18(5.24) | <.001 |
|  | Control | 45.10(6.44) |  |
| 1 | ITT | 49.78(5.05) | .001 |
|  | Control | 45.10(6.44) |  |
| 2 | ITT | 49.74(5.07) | .001 |
|  | Control | 45.10(6.44) |  |
| 3 | ITT | 49.79(5.04) | .001 |
|  | Control | 45.10(6.44) |  |
| 4 | ITT | 49.75(5.06) | .001 |
|  | Control | 45.10(6.44) |  |
| 5 | ITT | 49.83(5.04) | .001 |
|  | Control | 45.10(6.44) |  |

**Table4. Pre- and post-Imputation estimates of FACT-B and between-group differences at 3 months postintervention.**

| **Number** | **Group** | **Mean(SD)** | ***P* value** |
| --- | --- | --- | --- |
| Original data | ITT | 124.91(9.02) | .001 |
|  | Control | 113.50(11.20) |  |
| 1 | ITT | 122.77(8.66) | <.001 |
|  | Control | 113.50(11.20) |  |
| 2 | ITT | 122.86(8.57) | <.001 |
|  | Control | 113.50(11.20) |  |
| 3 | ITT | 122.74(8.70) | <.001 |
|  | Control | 113.50(11.20) |  |
| 4 | ITT | 122.82(8.61) | <.001 |
|  | Control | 113.50(11.20) |  |
| 5 | ITT | 122.83(8.60) | <.001 |
|  | Control | 113.50(11.20) |  |

Based on Table 1 to 5, the results from the original data without imputation, the five imputed datasets, and the pooled analysis all indicate that the primary and secondary outcomes are consistent with the results obtained using the EM imputation method under the assumption of MCAR. Therefore, the data imputation mechanism is reliable.

Reference:

[1] Jiang LL, Wang XY, Xu JH, Wu YW, Hu Y. Construction of an intelligent interactive nursing information support system and its application in young breast cancer survivors. Chin J Nurs. 2023;58(6):654-661. doi: 10.3761/j.issn.0254-1769.2023.06.002
